# Metagenomics insights into responses of rhizobacteria and their alleviation role in licorice allelopathy

**DOI:** 10.1186/s40168-023-01511-3

**Published:** 2023-05-22

**Authors:** Yang Liu, Hao Wang, Xun Qian, Jie Gu, Weimin Chen, Xihui Shen, Shiheng Tao, Shuo Jiao, Gehong Wei

**Affiliations:** 1 State Key Laboratory of Crop Stress Biology for Arid Areas, Shaanxi Key Laboratory of Agricultural and Environmental Microbiology, College of Life Science, Northwest A&F University, 3 Taicheng Road, Yangling, Shaanxi 712100 People’s Republic of China; 2 Interdisciplinary Research Center for Soil Microbial Ecology and Land Sustainable Productivity in Dry Areas, Northwest A&F University, Yangling, Shaanxi 712100 People’s Republic of China; 3 College of Natural Resources and Environment, Northwest A&F University, Yangling, Shaanxi 712100 People’s Republic of China

**Keywords:** Autotoxicity, Allelochemical, Rhizosphere, Continuous cropping obstacle, *Glycyrrhiza uralensis* Fisch.

## Abstract

**Background:**

Allelopathy is closely associated with rhizosphere biological processes, and rhizosphere microbial communities are essential for plant development. However, our understanding of rhizobacterial communities under influence of allelochemicals in licorice remains limited. In the present study, the responses and effects of rhizobacterial communities on licorice allelopathy were investigated using a combination of multi-omics sequencing and pot experiments, under allelochemical addition and rhizobacterial inoculation treatments.

**Results:**

Here, we demonstrated that exogenous glycyrrhizin inhibits licorice development, and reshapes and enriches specific rhizobacteria and corresponding functions related to glycyrrhizin degradation. Moreover, the *Novosphingobium* genus accounted for a relatively high proportion of the enriched taxa and appeared in metagenomic assembly genomes. We further characterized the different capacities of single and synthetic inoculants to degrade glycyrrhizin and elucidated their distinct potency for alleviating licorice allelopathy. Notably, the single replenished N (*Novosphingobium resinovorum*) inoculant had the greatest allelopathy alleviation effects in licorice seedlings.

**Conclusions:**

Altogether, the findings highlight that exogenous glycyrrhizin simulates the allelopathic autotoxicity effects of licorice, and indigenous single rhizobacteria had greater effects than synthetic inoculants in protecting licorice growth from allelopathy. The results of the present study enhance our understanding of rhizobacterial community dynamics during licorice allelopathy, with potential implications for resolving continuous cropping obstacle in medicinal plant agriculture using rhizobacterial biofertilizers.

Video Abstract

**Supplementary Information:**

The online version contains supplementary material available at 10.1186/s40168-023-01511-3.

## Background

Allelopathy was first put forward in 1937 by Molisch, who defined it as interactions among plants and/or microorganisms [1]. Autotoxicity is a unique form of allelopathy whereby a plant releases compounds (allelochemicals) into the environment that adversely affects its growth and development [2, 3]. Generally, allelopathy refers to allelopathic autotoxicity and is mainly driven by allelochemicals, most of which are secondary metabolites of plants, such as phenols and terpenoids [4]. Allelochemicals may be introduced into the soil environment by root exudation, volatilization, decomposition, and leaching during plant growth [5, 6]. Long-term selection under cultivation increases root secondary metabolite contents in medicinal plants, which makes plants more likely to release allelochemicals through root exudation. In addition, allelochemicals secreted by medicinal plants are almost homologous to their root secondary metabolites [3]. Therefore, compared to general crops, medicinal plants are more likely to exhibit allelopathic autotoxicity. Allelopathy is widely observed in agricultural ecosystems, such as monoculture, rotation, intercropping, and continuous cropping systems [7]. In recent years, in the wake of increasing demand for medicinal materials, the area under continuous cropping cultivation has increased. However, continuous cropping obstacle has emerged as a major problem limiting the agricultural production of Chinese traditional medicine [8], with allelopathic autotoxicity being the major factor causing the replant problem, which negatively affects the plant growth, yield, and quality [9, 10]. Consequently, effective strategies of addressing the continuous cropping obstacle are required.

As we all known, the rhizosphere is a hotspot of microbial diversity and activity in soils. It provides a microhabitat for diverse microorganisms under the influence of root exudates within narrow spaces in soil [11]. In addition, plants are exposed to various biotic and abiotic factors simultaneously, and their rhizospheres host microbes with diverse ecological functions [12, 13]. Numerous studies have reported that rhizosphere microbial community is closely linked to plant performance, including plant nutrition, plant growth, disease suppression, and abiotic stress resistance [14–16]. Furthermore, recently, some studies have reported that allelopathy are very complex rhizosphere biological processes involving chemical recognition and signal transduction between donor and recipient plants [4, 17, 18]. However, our understanding of the associations among rhizosphere microbial communities and autotoxic allelochemicals remains limited. The rhizosphere soil has been reported to be the largest source of allelochemicals under continuous cropping systems [19, 20]. Some studies have investigated the effects of allelochemicals on cucumber rhizosphere soil microbial community composition by simulating plant allelopathy following exogenous allelochemical supplementation [21, 22]. Moreover, the responses of rhizosphere soil microbial communities to artificially applied root exudates and replant problem of *Radix pseudostellariae* have been investigated [23]. In addition, the study to identify autotoxic substances and their activities in licorice rhizosphere soil firstly reported glycyrrhizin as the most powerful allelochemical involved in the replant failure of licorice [2]. Nevertheless, the effects of allelochemicals on licorice rhizobacterial communities have not been explored comprehensively.

Over the last decade, multi-omics sequencing technologies have been extensively applied in rhizosphere microbial community research [24–26]. An increased understanding of sequencing data could facilitate the comprehensive exploration of rhizosphere microbial community dynamics. Advances in the field of metagenomics have enabled the binning of genomes of individual community members in complex environments [27, 28]. The metagenomic sequencing has also been employed to better understand the diversity of secondary metabolite biosynthesis genes in soil bacterial communities [29] and to accurately identify carbohydrate and secondary metabolite transport and metabolism pathways correlated with bacterial enrichment in the sorghum rhizosphere under drought [30]. In spite of culture-independent approaches being able to provide greater information on the diversity and potential functions of microbial communities [31, 32], culture-based studies with isolation and purification technologies are still the guarantee to explore the function of rhizosphere microbial communities. Moreover, the potential functions of artificial synthetic communities composed of diverse bacteria have also been identified based on a combination of sequencing data and inoculation experiments in rice, maize, tomato, and garlic [33–36]. Numerous studies have demonstrated allelochemical degradation by microbes; for example, *Phomopsis liquidambari* degrades phenolic acid [37] and *Pseudomonus putida* degrades *p*-coumaric acid, with positive effects on bamboo growth [38]. However, allelochemical degradation by single or multiple rhizobacteria has not been investigated in licorice. Furthermore, no information is available on the metabolic pathways of allelochemicals in rhizobacterial communities of licorice.

Licorice (*Glycyrrhiza uralensis* Fisch.) is a perennial herbaceous leguminous plant and one of the most important traditional Chinese herbs [39]. Its roots and rhizomes contain two major secondary metabolites, glycyrrhizin and liquiritin [40], which have antitumorigenic and antioxidant properties, and have been used widely in tobacco and candy, and as medicine and food, globally [41, 42]. It is also an important cash crop in Northwest China [43]. High demand and low supply have led to continuous cropping of the plant. Considering the increased occurrence of continuous cropping obstacle in licorice, screening microbes for their potential ability to degrade allelochemicals in the rhizosphere could facilitate alleviation of the licorice replant problem and could not only enhance licorice production but also sustainable land use.

Consequently, in the present study, we used a combination of omics sequencing approaches and pot inoculation experiments (1) to investigate variations in licorice plant and soil characteristics, as well as rhizobacterial diversity and function under exogenous allelochemical addition, and (2) to elucidate the effects of rhizobacterial inoculants on licorice seedling performance. Accordingly, we hypothesized that (1) specific rhizobacteria could be enriched by exogenous allelochemical addition and (2) the enriched taxa could potentially degrade exogenous allelochemicals and alleviate licorice allelopathy.

## Results

### Variations in licorice plant and soil characteristics under allelochemical addition

**Fig. 1:**
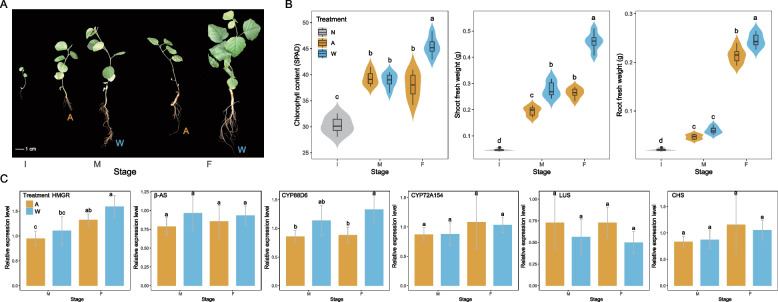
Licorice seedling phenotypes and the expression profiles of major secondary metabolite synthesis genes in root under exogenous glycyrrhizin addition. **a** Licorice seedling performance; **b** violin plots of licorice seedling chlorophyll contents, shoot and root fresh weights; **c** bar plots of expression levels of major secondary metabolite synthesis genes in licorice root among different sampling stages and treatments. I, initial sampling stage; M, middle sampling stage; F, final sampling stage; A, allelochemical treatment; W, water treatment; N, no treatment in initial samples. Different letters indicate significant differences (*P* < 0.05; one-way ANOVA, Tukey’s HSD test)

Plant performance was obviously affected by the exogenous allelochemical (glycyrrhizin) (Fig. 1a). Chlorophyll content in fresh leaves was significantly (*P* < 0.05) higher in the water treatment than in the allelochemical treatments at the final sampling stages. Fresh shoot and root weights exhibited similar trends, with higher values in the water treatment than in the allelochemical treatments across middle and final stages. Moreover, all plant phenotypic indices increased gradually with plant development from the initial to the middle and final stages (Fig. 1b). Meanwhile, according to qRT-PCR analysis data, allelochemical treatment suppressed the levels of expression of glycyrrhizin synthesis genes (*HMGR*, *β-AS*, *CYP88D6*) and enhanced the levels of expression of lupeol synthesis gene (*LUS*) across the middle and final stages. The levels of expression of *CYP72A154* and *CHS* were promoted by exogenous glycyrrhizin addition at the final stage (Fig. 1c). Generally, licorice development was impeded by exogenous glycyrrhizin to some extent, considering the plant phenotypes and expression profiles of major secondary metabolite synthesis genes in licorice root that were observed.

Soil characteristics varied in the course of plant development and were clearly elucidated between two soil compartments (bulk and rhizosphere soils) (Table 1). Rhizosphere soil pH increased significantly along with licorice growth, with higher values under allelochemical treatment. SWC was significantly higher in the water treatment than in the allelochemical treatments in the two compartments. SOM and TC values were significantly higher under allelochemical treatment than under the water treatment; furthermore, higher values were observed in bulk soil compared to rhizosphere soil, despite a lack of significant difference in most soil properties (TN, TP, TK, AN) among different stages and treatments. Soil AN, AP, and AK declined gradually in the course of licorice growth, and AK in rhizosphere soil had significantly higher values under allelochemical treatment than under the water treatments. Additionally, there were no significant differences in the activities of two soil enzymes between the two soil compartments, and their activities were generally inhibited by exogenous glycyrrhizin addition.

**Table 1:** Soil properties and enzymatic activities of two soil compartments between distinct stages and treatments

Compartment	Stage	Treatment	pH	SWC	SOM	TC	TN	TP	TK	AN	AP	AK	β-G	DHA
BS	I	-	8.8 ± 0.01 c	11.23 ± 0.53 f	5.45 ± 0.17 b	20.65 ± 0.03 f	0.34 ± 0.03 a	0.65 ± 0.01 ab	14.01 ± 0.17 ab	12.35 ± 0.64 a	6.04 ± 0.4 b	105 ± 1.41 d	117.05 ± 34.33 a	36.79 ± 4.15 a
	M	A	8.91 ± 0.01 b	12.7 ± 0.36 d	5.97 ± 0.19 a	20.91 ± 0.1 de	0.34 ± 0.04 a	0.66 ± 0.01 ab	13.92 ± 0.12 ab	12.02 ± 0.8 ab	6.6 ± 0.91 b	110.67 ± 0.52 ab	102.64 ± 28.57 a	35.92 ± 1.82 a
		W	8.66 ± 0.02 de	16.3 ± 0.26 b	5.44 ± 0.28 b	20.73 ± 0.04 ef	0.34 ± 0.03 a	0.67 ± 0 a	13.93 ± 0.07 ab	12.43 ± 1.06 a	11.66 ± 4.6 a	109 ± 0.89 ac	127.31 ± 28.01 a	35.29 ± 2.8 a
	F	A	8.96 ± 0.04 ab	17.21 ± 0.2 a	6.01 ± 0.17 a	21 ± 0.06 cd	0.38 ± 0.03 a	0.65 ± 0.01 ab	13.74 ± 0.4 ab	10.5 ± 0.49 c	5.45 ± 0.52 b	106 ± 3.35 cd	102.64 ± 17.88 a	34.14 ± 2.84 a
		W	8.6 ± 0.01 e	12.51 ± 0.34 d	5.43 ± 0.19 b	20.72 ± 0.11 f	0.36 ± 0.02 a	0.65 ± 0.01 ab	13.56 ± 0.49 b	9.77 ± 0.74 c	5.95 ± 0.41 b	97.83 ± 1.17 e	118.13 ± 30.27 a	34.67 ± 3.85 a
RS	I	-	8.64 ± 0.04 e	14.5 ± 0.26 c	5.8 ± 0.06 a	20.9 ± 0.02 de	0.37 ± 0.02 a	0.65 ± 0 ab	13.72 ± 0.36 ab	12.23 ± 0.58 a	6.32 ± 0.41 b	106.33 ± 1.97 cd	114.7 ± 27.72 a	31.69 ± 1.56 a
	M	A	8.92 ± 0.01 b	12.76 ± 0.28 d	6.04 ± 0.16 a	21.23 ± 0.19 ab	0.37 ± 0.01 a	0.66 ± 0.01 ab	13.76 ± 0.22 ab	12 ± 0.68 ab	8.51 ± 2.39 ab	111.67 ± 2.58 a	99.22 ± 21.03 a	35.02 ± 3.04 a
		W	8.74 ± 0.16 cd	17.18 ± 0.37 a	6.09 ± 0.14 a	20.96 ± 0.06 cd	0.36 ± 0.02 a	0.64 ± 0.01 b	14.16 ± 0.18 a	12.43 ± 0.87 a	6.71 ± 1.24 b	107 ± 1.79 bcd	111.46 ± 21.19 a	31.64 ± 1.65 a
	F	A	9.03 ± 0.01 a	11.86 ± 0.17 e	6.05 ± 0.18 a	21.37 ± 0.16 a	0.36 ± 0.01 a	0.66 ± 0.01 ab	13.7 ± 0.24 ab	9.35 ± 0.38 c	7.16 ± 1.11 b	105 ± 2.45 d	130.55 ± 27.33 a	33.64 ± 1.88 a
		W	8.75 ± 0.01 cd	14.06 ± 0.25 c	5.87 ± 0.09 a	21.1 ± 0.02 bc	0.36 ± 0.01 a	0.65 ± 0.01 ab	14.02 ± 0.35 ab	10.72 ± 0.97 bc	5.57 ± 0.18 b	88 ± 1.9 f	133.07 ± 30.26 a	35.48 ± 3.97 a
Group	F_values		45.92	291.40	15.54	34.62	2.17	2.73	2.41	15.00	6.67	75.02	1.18	2.04
	*P*_values		** < 0.001**	** < 0.001**	** < 0.001**	** < 0.001**	** < 0.05**	** < 0.05**	** < 0.05**	** < 0.001**	** < 0.001**	** < 0.001**	> 0.05	> 0.05

Values within the same column followed by different letters indicate significant differences (*P* < 0.05; one-way ANOVA, Tukey’s HSD test). Bold *P*-values indicate significant differences (*P* < 0.05)

*BS* bulk soil, *RS* rhizosphere soi, *I* initial sampling stage, *M* middle sampling stage, *F* final sampling stage, *A* allelochemical treatment, *W* water treatment, *pH* soil pH, *SWC* soil water content, *SOM* soil organic matter, *TC* total carbon, *TN* total nitrogen, *TP* total phosphorus, *TK* total potassium, *AN* available nitrogen, *AP* available phosphorus, *AK* available potassium, *β-G* β-glucuronidase, *DHA* dehydrogenase

### Varied rhizobacterial diversity and function under allelochemical addition

**Fig. 2:**
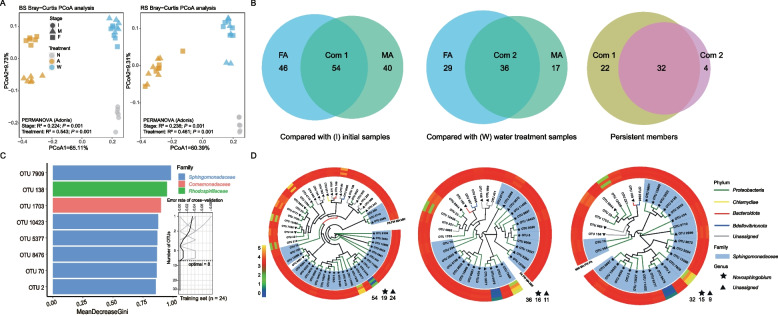
Variations in rhizobacterial communities under exogenous glycyrrhizin addition. **a** Principal coordinate analysis of rhizobacterial communities between two sampling compartments (BS, bulk soil; RS, rhizosphere soil) among different sampling stages and treatments. **b** Venn diagrams of enriched and persistent taxa between different treatments. I, initial sampling stage; M, middle sampling stage; F, final sampling stage; A, allelochemical treatment; W, water treatment. N, no treatment in initial samples. **c** The top eight bacterial families were identified using random-forest classification of the relative abundance of the persistent rhizobacterial taxa in allelochemical and water treatments. Bioindicators are ranked in descending order of importance to the accuracy of the model. The inset represents tenfold cross-validation error as a function of the number of input families used to differentiate allelochemical and water treatment rhizobacteria in order of variable importance. **d** Phylogenetic trees and relative abundance heatmaps of corresponding enriched and persistent taxa

Shannon diversity and ACE indexes exhibited similar trends, which varied significantly between distinct treatments, with higher values in the water treatments. Rhizobacterial community had higher diversity than bulk soil diversity (Table 2). In addition, community composition was significantly shaped by stages and treatments (*P* = 0.001). Stage had greater effects on the rhizobacterial composition (Adonis: *R*^2^ = 0.236) than bulk soil community composition (*R*^2^ = 0.224); on the contrary, the treatment had greater effects on bulk soil community composition (*R*^2^ = 0.543) than on rhizosphere soil community composition (*R*^2^ = 0.461) (Fig. 2a). Furthermore, most of the rhizobacterial taxa were co-enriched in allelochemical treatments across middle and final stages. Persistent taxa were selected based on intersections of co-enriched taxa that were derived from different groups (Fig. 2b). Afterward, eight bioindicator taxa were derived from the above persistent taxa based on random forests analyses, in which OTU 7909 had the highest explanatory degree despite its lower relative abundance. The bioindicators were mostly annotated as *Sphingomonadaceae* at the family level (Fig. 2c), and most of the specific enriched taxa were annotated as *Novosphingobium* genus, which belong to *Sphingomonadaceae* family and *Proteobacteria* phylum (Fig. 2d). Additionally, OTU 7909 was identified as a keystone taxon (SM Fig. S1A), and most of peripherals and keystone nodes in the network were assigned to the *Sphingomonadaceae* family (SM Fig. S1B). Overall, exogenous glycyrrhizin addition evidently affected rhizobacterial alpha and beta diversity and facilitated the recruitment of specific taxa that were partially defined as bioindicators.

**Table 2:** Bacterial alpha diversity of two soil compartments between distinct stages and treatments

Compartment	Stage	Treatment	Shannon	ACE
BS	I	-	8.88 ± 0.09 a	2190.36 ± 71.13 a
	M	A	6.03 ± 0.71 c	1754.84 ± 100.19 c
		W	8.9 ± 0.18 a	2152.14 ± 358.05 a
	F	A	6.89 ± 0.26 b	1769.51 ± 125.52 bc
		W	8.88 ± 0.1 a	2123.76 ± 207.69 ab
RS	I	-	9.04 ± 0.04 a	2098.6 ± 154.88 ac
	M	A	6.99 ± 0.31 b	1886.87 ± 95.11 ac
		W	9.02 ± 0.18 a	2070.9 ± 154.46 ac
	F	A	7.44 ± 0.54 b	1912.45 ± 226.3 ac
		W	8.96 ± 0.11 a	2174.43 ± 243.07 a
Group	*F*_values		73.74	4.54
	*P*_values		** < 0.001**	** < 0.001**

Values within the same column followed by different letters indicate significant differences (*P* < 0.05; one-way ANOVA, Tukey’s HSD test). Bold *P*-values indicate significant differences (*P* < 0.05)

*BS* bulk soil, *RS* rhizosphere soil, *I* initial sampling stage, *M* middle sampling stage, *F* final sampling stage, *A* allelochemical treatment, *W* water treatment

**Fig. 3:**
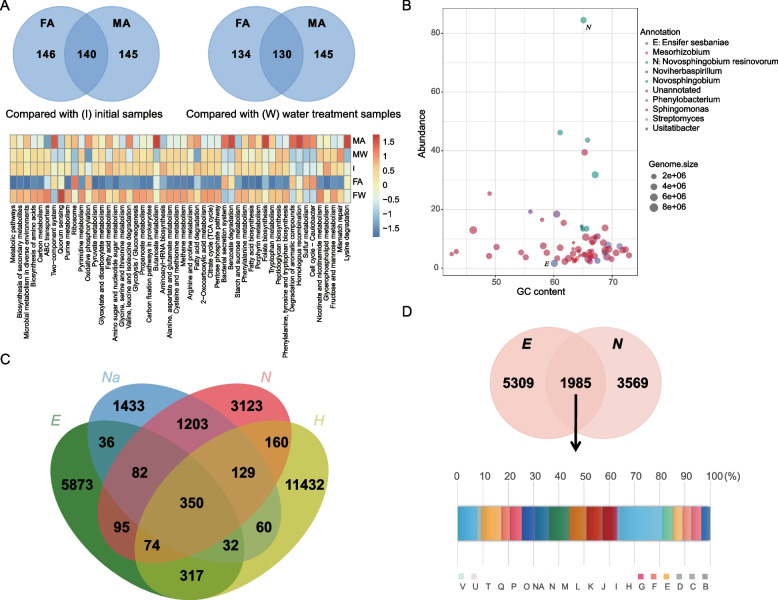
Metagenomic analyses of rhizobacterial communities and genomic information of four isolates. **a** Venn diagram showing the gene numbers with differential abundance (measured in TPM [transcripts per million]) and heatmap showing the relative abundance of the differentially enriched functional genes (top 50) among different groups. **b** Genome features of binned genomes:* x*-axis denotes the GC content (%) of the genome and *y*-axis denotes the abundance of the genome in the metagenome.** c** Pan-genome statistics of four strains: E, *Ensifer sesbaniae*; Na, *Novosphingobium arvoryzae*; N, *Novosphingobium resinovorum*; H, *Hydrocarboniphaga effuse.*** d** Functional enrichment of core genomes of strains E and N. The function of each gene was assigned using Clusters of Orthologous Groups (COG) categories. V, defense mechanisms; U, intracellular trafficking, secretion, and vesicular transport; T, signal transduction mechanisms; Q, secondary metabolites biosynthesis, transport, and catabolism; P, inorganic ion transport and metabolism; O, posttranslational modification, protein turnover, chaperones; NA, unannotated; N, cell motility; M, cell wall/membrane/envelope biogenesis; L, replication, recombination, and repair; K, transcription; J, translation, ribosomal structure, and biogenesis; I, lipid transport and metabolism; H, coenzyme transport and metabolism; G, carbohydrate transport and metabolism; F, nucleotide transport and metabolism; E, amino acid transport and metabolism; D, cell cycle control, cell division, chromosome partitioning; C, energy production and conversion; B, chromatin structure and dynamics

Metagenomic sequencing was used to further explore functional variations in rhizobacterial communities. Genes involved in 146 and 145 pathways were significantly enriched (Log [fold change] > 1, *P* < 0.05) following allelochemical treatment in the final and middle stages, respectively, when compared with those in the initial stage. Moreover, considerable overlaps were observed between the two differentially expressed gene sets (Fig. 3a). Similar observations were found in comparisons with the water treatment. Only slightly higher abundance of genes involved in degradation of cyclic compounds was observed (Fig. 3a). Furthermore, the reconstructed metagenome had 74 well-assembled genomes (taxa). The GC contents of the genomes ranged from 26.4 to 72.9%. The assemblage taxa were generally assigned to the genera *Mesorhizobium*, *Noviherbaspirillum*, *Novosphingobium*, *Phenylobacterium*, *Sphingomonas*, *Streptomyces*, and *Usitatibacter*, notably, with the *Novosphingobium* genus having the greatest abundance (Fig. 3b).

### Isolation and construction of inoculants

To explore the functions of the above enriched and assembled rhizobacteria, attempts were made to cultivate the enriched rhizobacteria using special screening medium. After five periods of subculture in the screening solution and dilution plate coating on agar-solidified medium, four strains were isolated by twice colony purifications. Additionally, the isolates were designated as *Ensifer sesbaniae* (E), *Novosphingobium arvoryzae* (Na), *Novosphingobium resinovorum* (N), and *Hydrocarboniphaga effuse* (H)*.* Afterward, the specific isolates were cultured in liquid screening medium to investigate their metabolites and rates of allelochemical degradation using LC–MS/MS analyses. The single N isolate had the highest degradation rate (87.94%) in pure culture followed by E, Na, and H (Table 3). However, the degradation metabolites were similar among the isolates, including Ginsenoside Rh, Ginsenoside Rh2, Betulinic acid, Oleanolic acid, and Ursolic acid, in addition to some Sterides, including Cholic acid, Ruscogenin, and Senegenin.

**Table 3:** Allelochemicals degradation rate of isolated strains

Isolates (species)	Degradation rate (%)
***Ensifer sesbaniae* (E)**	**76.68**
*Novosphingobium arvoryzae* (Na)	60.71
***Novosphingobium resinovorum* (N)**	**87.94**
*Hydrocarboniphaga effuse* (H)	36.96
**Synthetic communities (S)**	**92.95**

Bold fonts indicate the selected strains for finally constructing synthetic communities and for further experiments. Synthetic communities indicate mixtures of equal proportions of *Ensifer sesbaniae and Novosphingobium resinovorum* isolates

Furthermore, genome-wide sequencing was carried out and specific primers for the four isolates were designed. The core genes of the isolates were observed to be mostly associated with the housekeeping functions, such as ribosome assembly, DNA replication, transcription, and translation (SM Table S4). Despite no cyclic compound degradation genes being observed in their core genomes, a set of accessory and specific genes in the shell genomes were assigned in the cyclic compound degradation. Notably, the N and E isolates were associated with more exogenous substances biodegradation and metabolism genes than the other isolates (Fig. 3c, d). In addition, their genomic information of average nucleotide identity shared higher similarities (N: 84.4%; E: 84.2%) with the assembly genomes derived from metagenome binning. Moreover, there was no nutritional competition and antagonism between the two isolates based on plate culture experiments (SM Fig. S2). Last but not least, we selected and combined the two rhizobacterial isolates (N + E) as synthetic inoculants (S) due to (1) their enrichment in rhizosphere based on prior omics sequencing and analysis, (2) their relatively high allelochemical degradation rates, and (3) their genomic features. Additionally, the allelochemical degradation rate of synthetic inoculants was as high as 92.95% in the mixed culture (Table 3).

### Variations in licorice performance under rhizobacterial inoculants

**Fig. 4:**
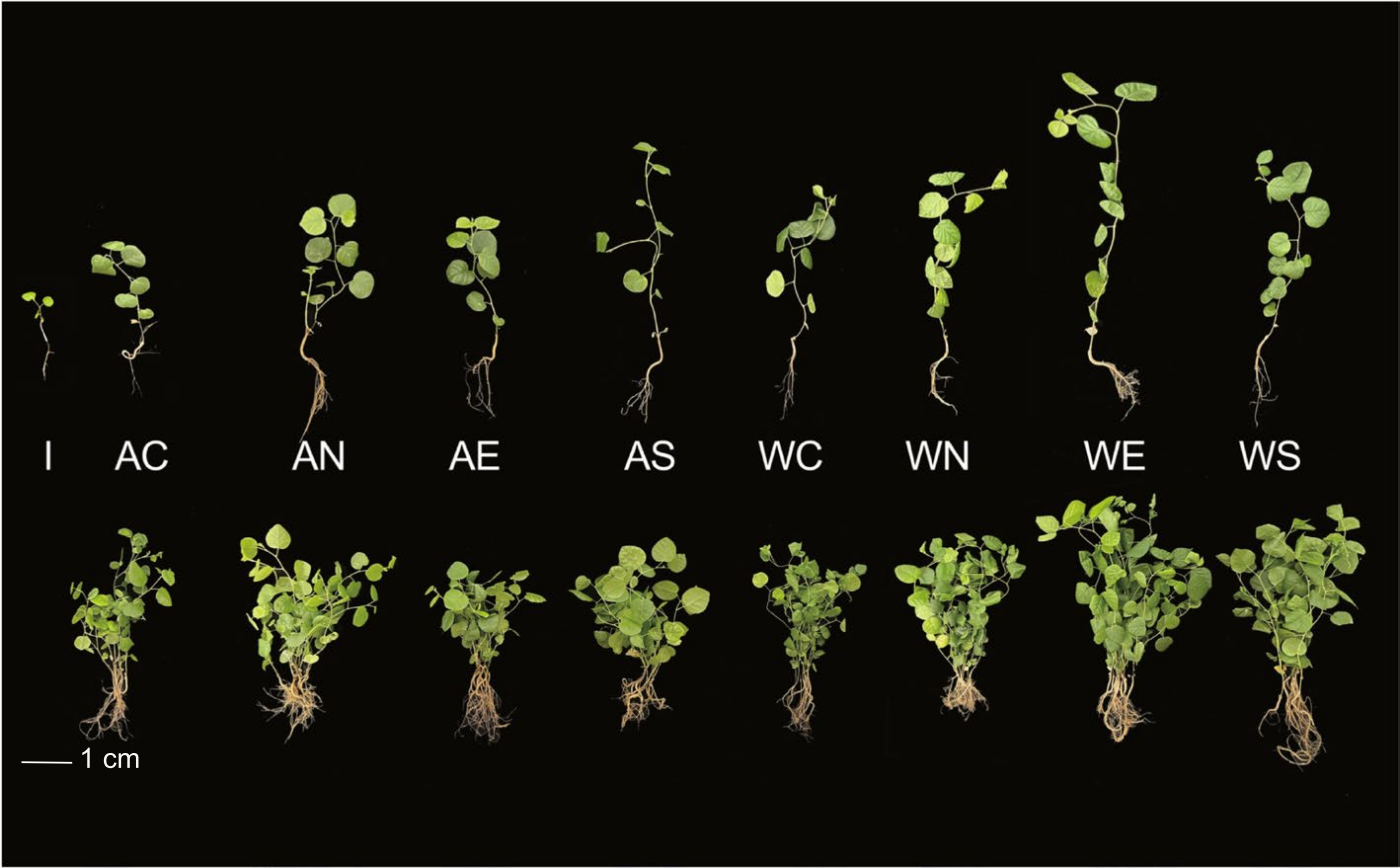
Licorice seedling performance under distinct inoculants and exogenous glycyrrhizin addition. I, initial sampling stage; A, allelochemical treatment; W, water treatment; C, control: no inoculants, N, *Novosphingobium resinovorum* inoculants; E, *Ensifer sesbaniae* inoculants; S, synthetic inoculants. The upper and lower plants represented single and clustered seedlings at the same sampling stage, respectively

Subsequently, we conducted pot experiments to verify the effects of the above single and synthetic rhizobacterial inoculants on licorice seedling performance. The inoculants were deemed rhizobacterial replenishments considering they originated from and existed in the original experiment soils. Allelochemical treatment inhibited the licorice seedling development based on the plant phenotypes (Table 4 and Fig. 4). Higher seedling growth indices were observed in all inoculants than in no inoculants (C). Furthermore, the single N strain inoculated had the highest seedling growth index values under allelochemical treatments; however, the single E strain inoculated had the highest values in water treatment. Moreover, shoot fresh weight was significantly varied (*F* = 288.04, *P* < 0.001) among different groups. Additionally, the synthetic inoculants (S) had lower seedling growth indices in allelochemical treatment than in water treatment. Simultaneously, the expression profiles of major secondary metabolite synthesis genes in licorice root were investigated. The inhibition effects of exogenous allelochemical on glycyrrhizin synthesis genes were alleviated to a certain extent by the inoculants. Particularly, the single N and E inoculants had promoted the expression profiles of glycyrrhizin synthesis genes (*HMGR*, *β-AS*, *CYP88D6*) in both allelochemical and water treatments, respectively (Table 5). But the single inoculant of N strain had strong promotion effects on lupeol synthesis genes (*CYP72A154*, *LUS*, *CHS*) in water treatment.

**Table 4:** Licorice seedling performance under combined effects of allelochemical addition and rhizobacterial inoculants

Treatment	Inoculation	Chlorophyll content (SPAD)	Shoot fresh weight (g)	Root fresh weight (g)
-	I	29.98 ± 1.11 d	0.05 ± 0.003 f	0.02 ± 0.002 b
A	C	32.56 ± 1.494 c	0.24 ± 0.037 d	0.04 ± 0.004 b
	N	36.51 ± 1.742 ab	0.33 ± 0.042 c	0.13 ± 0.013 ab
	E	34.29 ± 2.157 bc	0.25 ± 0.032 d	0.05 ± 0.005 b
	S	36.41 ± 1.507 ab	0.26 ± 0.028 d	0.07 ± 0.007 ab
W	C	35.4 ± 2.403 ab	0.33 ± 0.032 c	0.07 ± 0.003 ab
	N	37.27 ± 0.935 a	0.65 ± 0.071 b	0.13 ± 0.012 ab
	E	37.59 ± 1.391 a	0.82 ± 0.05 a	0.18 ± 0.008 a
	S	36.94 ± 1.517 a	0.34 ± 0.031 c	0.12 ± 0.238 ab
Group	F_values	21.28	288.04	3.94
	*P*_values	** < 0.001**	** < 0.001**	** < 0.001**

Values within the same column followed by different letters indicate significant differences (*P* < 0.05; one-way ANOVA, Tukey’s HSD test). Bold *P*-values indicate significant differences (*P* < 0.05)

*A* allelochemical treatment, *W* water treatment, *I* initial sampling stage, *C* control: no inoculants, *N Novosphingobium resinovorum* inoculants, *E Ensifer sesbaniae* inoculants, *S* synthetic inoculants

**Table 5:** Expression profiles of major secondary metabolite synthesis genes in licorice root under combined effects of allelochemical addition and rhizobacterial inoculants

Treatment	Group	HMGR	β-AS	CYP88D6	CYP72A154	LUS	CHS
-	I	0.79 ± 0.2 a	1.17 ± 0.15 c	1.04 ± 0.07 c	1.02 ± 0.11 ab	1.11 ± 0.1 ab	0.99 ± 0.07 a
A	C	1.09 ± 0.27 a	1.8 ± 0.37 bc	1.65 ± 0.2 ac	0.86 ± 0.18 ac	0.26 ± 0.03 c	0.24 ± 0.08 b
	N	1.32 ± 0.15 a	3.08 ± 0.67 a	2.11 ± 0.4 a	0.67 ± 0.13 bc	0.25 ± 0.01 c	0.17 ± 0.01 b
	E	1.31 ± 0.29 a	2.41 ± 0.09 ab	1.84 ± 0.09 ab	0.63 ± 0.05 c	0.31 ± 0.08 c	0.14 ± 0.02 b
	S	0.79 ± 0.41 a	2.06 ± 0.15 ac	1.13 ± 0.1 c	0.61 ± 0.05 c	0.25 ± 0.04 c	0.15 ± 0.03 b
W	C	0.96 ± 0.12 a	1.71 ± 0.18 bc	1.12 ± 0.23 c	0.67 ± 0.15 ac	0.2 ± 0.03 c	0.18 ± 0.07 b
	N	0.84 ± 0.33 a	1.81 ± 0.6 bc	1.32 ± 0.5 bc	1.05 ± 0.17 a	1.3 ± 0.52 a	0.26 ± 0.08 b
	E	0.96 ± 0.15 a	2.16 ± 0.38 ac	1.36 ± 0.08 bc	0.75 ± 0.18 ac	0.62 ± 0.11 bc	0.2 ± 0.01 b
	S	0.86 ± 0.11 a	1.72 ± 0.46 bc	1.24 ± 0.06 bc	0.59 ± 0.11 c	0.34 ± 0.09 c	0.15 ± 0.02 b
Group	F_values	2.098	5.541	6.637	5.210	14.570	88.392
	*P*_values	0.091	**0.001**	** < 0.001**	**0.002**	** < 0.001**	** < 0.001**

Values within the same column followed by different letters indicate significant differences (*P* < 0.05; one-way ANOVA, Tukey’s HSD test). Bold *P*-values indicate significant differences (*P* < 0.05)

*A* allelochemical treatment, *W* water treatment, *I* initial sampling stage, *C* control: no inoculants, *N Novosphingobium resinovorum* inoculants, *E Ensifer sesbaniae* inoculants, *S* synthetic inoculants

Furthermore, allelochemical contents in rhizosphere soil following inoculation were lower than those with no inoculation in all treatments (SM Table S3). The lowest allelochemical contents were observed in the single N (34.94%) and E (20.19%) inoculants in the allelochemical and water treatments, respectively. In contrast, the highest allelochemical contents were observed in the synthetic inoculants (S) apart from no inoculants (C) in the water treatment. Notably, the numbers of E strain were the highest in the rhizosphere following supplementation of allelochemical treatment with N isolate and in the water treatment without inoculant (SM Fig. S3). In addition, N strain numbers were the highest in allelochemical treatment supplemented with N isolate and in water treatment supplemented with E isolate, excluding in the initial samples. Overall, the preliminary data above suggest that potential protection measures of the rhizobacterial inoculants against licorice are attributable to their colonization and allelochemical degradation capacities in rhizosphere soil.

## Discussion

Plant allelopathy has been simulated by exogenous addition of allelochemical in several previous studies [22, 23] and demonstrated based on variations in plant, soil, and rhizobacterial community characteristics under different treatments. Most of the enriched rhizobacteria in licorice under glycyrrhizin treatments in the present study were assigned to genus *Novosphingobium*, which was also re-assembled in the metagenomic analyses, and had been reported to have the capacity to degrade cyclic compounds in other environments [44, 45]. Correspondingly, some enriched functional genes were slightly involved in the degradation of cyclic compounds. The function was also observed in shell genomes of the enriched and isolated rhizobacteria in the present study. And the main chemical structure of glycyrrhizin is mainly composed of cyclic structures [2]. Therefore, we inferred that glycyrrhizin could be degraded by specific isolates. After the inoculation experiments, the degradation ability of inoculants for glycyrrhizin was confirmed by allelopathy alleviation in licorice, and optimal results were observed with the single N (*Novosphingobium resinovorum*) inoculant, supported by degradation and plant growth-promoting features of the genus reported in other studies [46, 47].

### Effects of exogenous glycyrrhizin on plant and soil characteristics

Allelochemical effects were enhanced by the exogenous addition of glycyrrhizin, which simulated allelopathic effects, as described in other studies [22, 23]. In the current study, licorice plant phenotypes were affected the most following glycyrrhizin addition, which is consistent with the findings of a former study [2]. Therefore, glycyrrhizin suppressed licorice growth compared to that in the water treatment. However, it did not restrict licorice temporal development. The results indicate that the concentration of exogenous glycyrrhizin was appropriate for licorice survival. According to previous studies, most of the key genes involved in glycyrrhizin synthesis have been successfully cloned and characterized [48, 49]. Therefore, based on our qRT-PCR analysis results, glycyrrhizin synthesis in licorice root was impeded by exogenous glycyrrhizin addition.

Furthermore, comparing to bulk soil, the rhizosphere soil was mostly under the influence of plant root exudates [19, 50]. Therefore, the soil properties were considerably affected by sampling compartments instead of exogenous glycyrrhizin. SOM and TC were enhanced in rhizosphere soil and under glycyrrhizin treatment because the exogenous glycyrrhizin and root exudates simultaneously provided the carbon sources. Notably, the pH of the glycyrrhizin solution was balanced by NaOH to avoid the direct acidic effects of glycyrrhizin; therefore, salt solution addition facilitated to elevation of alkalization degree in rhizosphere soil. In addition, most of the total soil nutrients were stable and were not affected by glycyrrhizin addition, which indicated that exogenous glycyrrhizin addition directly induced allelopathy rather than altered soil properties. Soil enzyme activities have been linked to polysaccharide degradation in a previous study [51]. Considering the basic skeleton of the chemical structure of glycyrrhizin is similar to those of some polysaccharides [2], accumulation of exogenous glycyrrhizin put great pressures on the metabolism of polysaccharides, thus leading to the relatively low enzyme activities in licorice rhizosphere soil.

### Differential enrichment of rhizobacteria and functional genes

In the present study, exogenous glycyrrhizin addition decreased the alpha diversity of rhizobacterial communities and altered community composition across different stages and compartments, to varying degrees, which were mostly attributed to the effects of plant root exudate and glycyrrhizin selection. Such findings are similar to the results of previous studies, where allelochemicals have been demonstrated to have the capacity to alter soil microbial community composition and diversity [22, 52]. Furthermore, in the present study, exogenous glycyrrhizin addition and licorice root exudates enrich specific rhizobacteria, and a proportion of the enriched taxa has been considered persistent taxa [53]. Additionally, the key bioindicator (OTU 7909) in the present study was derived from persistent taxa, and most of the enriched taxa and bioindicators were generally annotated as the *Novosphingobium* genus or *Sphingomonadaceae* family. Previous studies have demonstrated the degradation of cyclic compounds by *Novosphingobium* genus or *Sphingomonadaceae* family in distinct environments [44, 45, 47]. Considering the main chemical structure of glycyrrhizin is similarly composed of cyclic structures [2], we inferred that some enriched rhizobacteria were potentially able to degrade glycyrrhizin.

Functional changes were evaluated using metagenomic analyses. The compared method of differential enrichments of functions was similar with the selection approaches of rhizobacterial taxa, which confirmed the available relationships between the taxa and the corresponding functions. Only a few genes were involved in the degradation of cyclic compounds, which is mainly attributed to the screening threshold of differential functional genes and annotation methods of functional genes. The classification of metagenomic functions represented by COG, KEGG, and GO have contributed to distinct results for the same data based on previous studies [54]. Furthermore, dozens of genomes were assembled, in which one genome exhibited large similarity with the most highly enriched bioindicator, namely *Novosphingobium* genus. In addition, the *Novosphingobium* had the greatest abundance in the metagenomic assembly genomes (MAGs), indicating their potential capacity to degrade glycyrrhizin and adapt to the rhizosphere environment, in the present study.

### Alleviation effects of inoculants on licorice allelopathy

To verify the effects of enriched rhizobacteria on licorice development, four strains were isolated from the rhizosphere soil using the enrichment cultivation method in pure culture, as described in other studies [55, 56]. A few strains could survive in the special medium according to our improved method for re-enrichment of rhizobacteria. Subsequently, the isolates were identified as distinct species, with great similarity with the enriched or metagenomic assembly taxa, such as the N and E isolates. Such findings have also been reported in previous studies, demonstrating that functional strains revealed from omics analyses could be isolated using special methods, and be explored further [34, 57].

The glycyrrhizin degradation efficiency of the isolates in pure culture varied, according to their respective characteristics, which were further investigated based on their genomic information. As the complete glycyrrhizin degradation pathway has not yet been reported, and the features of strains similar to our isolates have been reported by previous studies [44, 45], the genes involved in the degradation of cyclic compounds were preliminarily predicted to be associated with glycyrrhizin degradation. In addition, the shell genomes of all the isolates were associated with cyclic compound degradation. However, no core genes were responsible for the function, indicating that the isolates utilize different sets of genes or even have co-catabolism strategies for cyclic compound degradation [58]. Moreover, the results of genome-wide sequencing were limited, since the complete and exact degradation route(s) of glycyrrhizin have not yet been characterized. Consequently, our future study will focus on the screening and identification of the distinct genes participating in glycyrrhizin catabolism. Notably, the N and E isolates largely possessed exogenous substance biodegradation and metabolism genes. In addition, they both appeared in the MAGs and had higher degradation efficiency, which led to their selection and use in formulation of synthetic inoculants for subsequent experiments.

Finally, inoculant experiments were carried out to investigate the effects of single and synthetic inoculants on licorice seedling performance. Most of the validation experiments for inoculants in previous studies have been based on sterilized substrates or soils [35, 36]; however, in the present study, the soils were originally collected from a continuous cropping licorice field, to maintain the soil properties and interactions between indigenous rhizobacteria and replenished inoculants. Moreover, exogenous glycyrrhizin addition was still executed after inoculation, because the allelopathy was persistent due to continuous cropping obstacle following the accumulation of allelochemicals [59]. Alleviation effects of inoculants on licorice allelopathy were obvious and distinct across different inoculants. The results indicated that distinct inoculants had the potential to degrade glycyrrhizin not only in pure culture but also in licorice rhizosphere soil.

In addition, different colonization rates in rhizosphere soil result from their differences in glycyrrhizin metabolism and tolerance, and the colonization density of inoculants is closely related to their function. Greater colonization density of inoculants may be more conducive to the competition for resources to ensure their own reproduction and functional features. It was also reported that the relative abundance of most rhizoplane-enriched bacteria is closely associated with disease resistance of citrus [55]. Notably, in the present study, the single N (*Novosphingobium resinovorum*) inoculate had the strongest allelopathy alleviation effects. Despite the single E (*Ensifer sesbaniae*) inoculant playing important roles in water treatment, potentially due to its nodulation ability [60], the colonization rate of the N inoculant was higher in the E inoculant treatment. The results demonstrated the positive effects of N inoculant on licorice performance with respect to allelochemical degradation and plant growth-promotion features, as reported in the genus in other studies [46, 47]. The N strains could degrade allelochemicals, which maybe lead to the improvement of the living environment of E strains and driving the colonization of E strains. Despite the E strains could degrade allelochemicals when the rhizosphere environment was suitable, this did not mean that they preferred to use it. Additionally, although synthetic inoculants have been reported to have greater effects in other studies [33, 61], they had lower alleviation effects compared to that of the single inoculant, in the present study. That resulted from the inoculants of N and E strains having complicated interactions with own and indigenous rhizobacterial communities, which should be paid more attention for further investigation of variations in rhizobacterial communities after inoculations in the future. The highest glycyrrhizin degradation rate in rhizosphere soil was observed under single inoculant treatments, despite the highest glycyrrhizin degradation rate being observed in S inoculants in pure culture. The results could be due to their discrepancy metabolism capacities under different environments, because different treatments generated varied rhizosphere environment and bacterial communities including the inoculants that originated from and existed in the original experiment soils. It also should be pointed that glycyrrhizin could affect processes that were not evaluated in the present study. Therefore, metabolomics analyses of soil and microorganisms should be incorporated in future studies to comprehensively reveal the detoxification mechanisms of inoculants for licorice allelopathy.

## Conclusions

In the present study, two pot experiments were performed to investigate the responses of licorice and its rhizobacterial communities to exogenous glycyrrhizin addition, and to elucidate the effects of specific isolated rhizobacteria on the licorice allelopathy. Licorice development was impeded by exogenous glycyrrhizin addition to some extent; however, soil characteristics were almost not affected by glycyrrhizin addition. In addition, glycyrrhizin apparently decreased rhizobacteria community alpha diversity and alter rhizobacterial community composition to varied degrees, across sampling stages and compartments. Moreover, specific rhizobacteria and functions associated with glycyrrhizin degradation were enriched in allelochemical treatments, and most of the taxa were generally assigned to the genus *Novosphingobium*, which was also assembled in the metagenomic analyses. Subsequently, four rhizobacterial isolates were purified from the above rhizosphere soil, and two were selected for use in developing synthetic inoculants. Furthermore, single and synthetic inoculants were added to the rhizosphere soil, and assay results showed that alleviation effects of inoculants on licorice allelopathy were obvious and different across various inoculants. Notably, the single N (*Novosphingobium resinovorum*) inoculant had the greatest effects, which highlighted the potential of harnessing such rhizobacteria for manipulating allelochemical degradation in continuous medicinal plant agricultural ecosystems. The results of the present study also enhance our understanding of the interactions between rhizobacterial communities and plant allelochemicals and provide a framework for managing the continuous cropping obstacle in medicinal plant agriculture from the perspective of rhizosphere microbial communities.

## Methods

### Experimental setup

Soils used in the present study were collected from the upper soil layer (0–20 cm) at three random sites in one field where licorice (*Glycyrrhiza uralensis* Fisch.) had been grown for 2 years in the Yuzhong North Mountains region (104° 18′–104° 19′ E, 36° 8′–36° 9′ N), in Lanzhou, Gansu province, northeast China [39]. The soil conditions are optimal for licorice growth. The collected soils were passed through a 2-mm mesh sieve to remove (plant) debris and stored for subsequent pot experiments.

The first pot experiment was performed to assess the effects of exogenous glycyrrhizin (allelochemical) on licorice plant and rhizobacterial community characteristics. Licorice seeds were surface-sterilized serially in 70% ethanol for 1 min, in 1% sodium hypochlorite solution for 10 min, and finally rinsed extensively in sterile water five times. Surface-sterilized seeds were placed on a plate with filter paper for 24 h at 4 °C and then were germinated at 28 °C in the dark. Three days later, the seedlings were transplanted into pots (8-cm bottom diameter, 10-cm top diameter, 10-cm height) containing 200 g of soil and maintained in a controlled growth chamber (30 °C day/20 °C night, 60–80% relative humidity, 16-h light/8-h dark, and 300 μmol/m^2^/s photosynthetically active radiation). Each pot originally contained four licorice seedlings. After 4 weeks, a single licorice seedling (two pieces of true leaf) was retained in each pot for further treatments. Previous studies have shown that some root exudates can be depleted rapidly after addition to the soil because of microbial decomposition and utilization [62]. Considering glycyrrhizin is a major root exudate and allelochemical in licorice [2], we added 2.5 mg/ml exogenous glycyrrhizin solution (EGS (2.5 g glycyrrhizin and 0.3 g sodium hydroxide [NaOH], pH 7.5, 25 °C) periodically to soil to maintain the desired concentration. The glycyrrhizin concentration in the field soil was 0.015 μmol g^−1^ soil [2]. After 1 week, licorice seedlings at the three-leaf stage were treated with 10 ml EGS (0.15 μmol g^−1^ soil) and 20-ml tap water once every 4 days as described in previous studies [22, 23]. The soil treated with tap water (30 ml) was used as the control. Soil water content was adjusted to approximately 60% water holding capacity every 4 days with tap water to maintain constant water content in pots. In total, there were 30 bulk soil samples (30 pots: 6 replicates [initial] + 2 treatments × 6 replicates × 2 stages [middle and final]) as well as 30 rhizosphere soil and plant samples (150 pots: 6 replicates × 5 pots/seedlings [initial] + 2 treatments × 6 replicates × 5 pots × 2 stages [middle and final]).

**Fig. 5:**
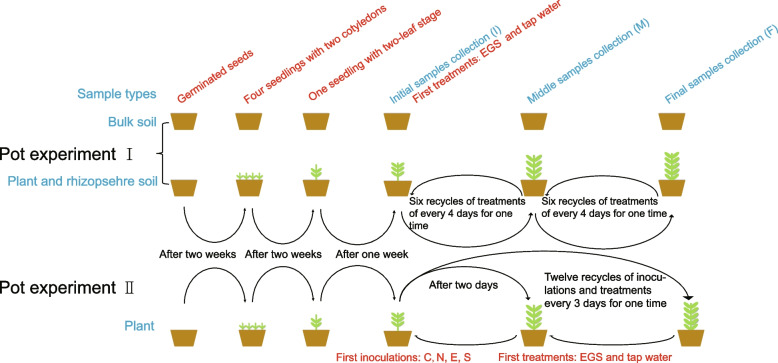
Experimental setup and sampling conditions. Red font, experimental treatments; blue font, sample types and stages. Pot experiment I, addition of exogenous allelochemical (glycyrrhizin) experiment; pot experiment II, inoculant and glycyrrhizin addition experiment

A second set of pot experiments was carried out to verify the effects of rhizobacterial inoculant on licorice performance under the following addition of exogenous glycyrrhizin to licorice pots in a growth chamber. Inoculation suspensions were prepared to have an OD_600nm_ of 0.08–1.0. The inoculant was poured near plant roots in pots at a density of 10^7^ cells/g soil. In the single inoculant treatments (N: *Novosphingobium resinovorum*; and E: *Ensifer sesbaniae*), 10-ml suspension was used. However, in the synthetic inoculant treatments (S: N + E), 5-ml suspensions of each of the two inoculants were used. The control treatment (C) was inoculated with 10-ml sterile water. After 2 days of inoculation, licorice seedlings were treated with 10-ml EGS and 10-ml tap water once every 3 days. The soils treated with tap water (20 ml) were used as the control. In total, there were 27 plant samples (81 pots: 3 replicates × 3 pots/seedlings [initial] + 4 inoculations × 2 treatments × 3 replicates × 3 pots). The experimental design and sampling stages are shown in detail in Fig. 5.

### Samples’ collection and processing

In the first pot experiment, six pots with the same amount of soil but without plants were used as controls and served as bulk soil (BS) samples. The rhizosphere soil (RS) samples were obtained using a routine sampling method [63]. The bulk and rhizosphere soils were sieved through a 2-mm mesh to remove visible roots, residues, and stones and then divided further into three subsamples. One subsample was used for the analysis of edaphic properties, including soil pH, soil water content (SWC), soil organic matter (SOM), total carbon (TC), total nitrogen (TN), total phosphorus (TP), total potassium (TK), available nitrogen (AN), available phosphorus (AP), and available potassium (AK) as previously described [64]. Another subsample was used for the analysis of enzyme activity, including β-glucuronidase (β-G) and dehydrogenase (DHA) activity, using an enzyme spin kit for soil (Keming Biological Co., Ltd., Suzhou, China) and a microplate reader, as described in a previous study [51]. The third subsample was used for DNA extraction. Briefly, total DNA was extracted from BS and RS samples (0.5 g each) using a Fast DNA® SPIN Kit (MP Biochemicals, Solon, USA) according to the standard manufacturers’ procedures. DNA concentration and purity were estimated using a Nanodrop 1000 spectrophotometer (Thermo Fisher Scientific, Waltham, MA, USA) and electrophoresis in 1% (w/v) agarose gels.

Chlorophyll fluorescence measurement was conducted on six leaves from each licorice seedling using a Plant Efficiency Analyzer (Hansatech Instruments Ltd., Norfolk, England). Before measurement, leaves were pretreated in the dark for 30 min. Licorice plant samples were then collected whole from the pot soil, and the plant roots were used to obtain RS. Shoot and root fresh weights were measured immediately after sample collection. Total RNA isolation from plant roots was performed using the MiniBEST Plant RNA Extraction Kit (Takara, Dalian, China) according to the manufacturer’s instructions. After the extraction, purification was carried out using MicroElute RNA Clean-Up Kit (Omega Biotek, USA) and DNase I (Takara Biotechnology Co. Ltd, Dalian, China) treatment. RNA concentration and purity were checked using a Nanodrop 1000 spectrophotometer (Thermo Fisher Scientific) and electrophoresis in 1% (w/v) agarose gels. Complementary DNA (cDNA) were synthesized from pretreated total RNA using a RevertAid First Strand cDNA Synthesis Kit (Thermo Fisher Scientific Inc., USA) according to the manufacturer’s instructions.

The expression profiles of major secondary metabolite synthesis genes were more accurate at RNA level, and the quantitative real-time PCR (qRT-PCR) analysis could provide a precise quantitative approach for detecting secondary metabolite contents in licorice root [48]. Thus, the levels of expression of glycyrrhizin and liquiritin biosynthesis genes were analyzed by qRT-PCR using a Quantstudio 6 Flex real-time PCR system (Thermo Fisher, Carlsbad, CA, USA) and SYBR Premix Ex Taq II (TaKaRa). Thermal cycling conditions were as follows: initial denaturation at 95 °C for 10 s, followed by 40 cycles at 95 °C for 15 s, 56 °C for 60 s, and 72 °C for 30 s. Data collection was performed at 72 °C. A melting curve was generated to monitor amplification specificity, and the procedure was as follows: 95 °C for 10 s, 60 °C for 60 s, 95 °C for 15 s, and 60 °C for 15 s. The primers used for gene amplification are listed in Table S1. qRT-PCR analysis was carried out with independent biological replicates and three technical replicates. Relative quantification of gene expression levels was performed using the comparative 2^−ΔΔCt^ method [65]. Purified RNA and RNA-free water were used as negative controls to preclude genomic DNA contamination and primer-dimer production. Expression values were normalized using two housekeeping genes, including *18 s ribosomal RNA* and *β-actin* [66].

### Amplicon sequencing and bioinformatics analyses

The hypervariable V4–V5 region of the 16S rRNA gene was selected for the amplification of bacterial sequences using the 515F (GTGCCAGCMGCCGCGGTAA)/907R (CCGTCAATTCCTTTGAGTTT) primer pair [50]. All polymerase chain reactions (PCR) were performed based on the procedures described in a previous study [67]. Triplicate PCR amplicons were pooled together and then mixed with a similar volume of 1 × loading buffer (containing SYB green). They were detected by electrophoresis in 2% (w/v) agarose gel. PCR products with bright bands between 400 and 450 bp were mixed in equal density ratios and purified using a GeneJET™ Gel Extraction Kit (Thermo Fisher Scientific). DNA sequencing libraries were generated using an Ion Plus Fragment Library Kit (48 rxns; Thermo Fisher Scientific). The library quality was assessed using a Qubit@ 2.0 Fluorometer (Thermo Fisher Scientific). Finally, the library was sequenced using an Ion S5^TM^XL platform (Thermo Fisher Inc., Waltham, MA, USA) [68] and 400-bp single-end reads were generated by Novogene Co., Ltd. (Beijing, China).

Low-quality sequences were sheared using Cutadapt [69]; QIIME pipeline (v1.7.0) and USEARCH tool were used for quality-filtering [70] and removal of chimeric sequences [71], respectively. The clean sequence reads were assigned to operational taxonomic units (OTUs) based on a similarity threshold of 97% using the UPARSE pipeline [72]. A representative sequence of each OTU was annotated with taxonomic information using the SILVA 132 database [73]. After removing the plastid sequences from the data, a total of 2,168,504 bacterial (36,142 ± 9244) quality reads were obtained (after filtering) from the 60 samples (30 samples per sampling compartment). Additionally, a total of 13,432 (bacterial: 1923 ± 278) OTUs were clustered. After homogenization, subsequent analysis was carried out based on a minimum of 19,908 bacterial reads per sample to ensure equal sampling effort across all samples. The alpha diversity indices (Shannon and ACE) of the bacterial communities were calculated using the “vegan” package in R (v3.6.3) [74] based on the rarefied OTU tables and their phylogenetic trees.

### Metagenomics sequencing and data processing

According to the preliminary results, rhizosphere soil samples were selected to further carry out metagenomics sequencing on an Illumina NovaSeq 6000 platform (Illumina, San Diego, CA). Raw reads were first converted to the fastq. format. The quality of each sample was checked using the FastQC tool; afterward, the adapters were removed using Trimmomatic (v0.39) in KneadData (v0.6.1) [75] using the following parameters: SLIDINGWINDOW:4:20 MINLEN:50. The metagenomics data were assembled using megahit [76]. The genes and other features of each assembled metagenome were annotated using Prokka (v1.14.5) [77] using the “—metagenome” tag. Gene function classification is represented by Clusters of Orthologous Groups (COG), Kyoto Encyclopedia of Genes and Genomes (KEGG), and Gene Ontology (GO) databases using eggNOG (evolutionary genealogy of genes: Non-supervised Orthologous Groups) [78]. The abundance of the genes was measured by TPM (transcripts per million) calculated by salmon software in metagenome mode. Metagenome binning was executed based on each separated sample using the MetaWRAP pipeline [79].

### Isolation and identification of inoculants

Inorganic salt solution (KH_2_PO_4_ 2.5 g, MgSO_4_·7H_2_O 0.2 g, FeSO_4_·7H_2_O 0.1 g, K_2_HPO_4_ 2.0 g, NH_4_NO_3_ 3.0 g in 1 L, pH 6.0 ~ 6.5, 25 °C) containing glycyrrhizin as the sole carbon source was used for all isolate screening, isolation, and culture experiments. Appropriate amounts of NaOH were added to the culture solutions to adjust the pH (7.5–8.0) of the special screening medium. Rhizosphere soil samples (10 g) were added to 90 ml of autoclaved water. Soil suspension (2 ml) was added to 20 ml (10%) screening medium containing 2.5 g l^−1^ of glycyrrhizin and incubated for 5 days at 30 °C with shaking (rotary shaker at 180 rpm). The first culture solution (2 ml) was transferred into new 20 ml of screening medium containing 3 g l^−1^ of glycyrrhizin and incubated for another 5 days. Afterward, the culture solution was continuously transferred into new screening media containing 4, 6, and 10 g l^−1^ of glycyrrhizin, three times. The purpose of the four transfers to fresh media was to dilute and reduce potential carbon sources from original rhizosphere soils. The fifth culture solution was inoculated onto an agar-solidified screening medium containing 10 g l^−1^ of glycyrrhizin. After 5 days of incubation at 30 °C, a single colony of each potential species was selected, suspended in autoclaved ddH_2_O, inoculated onto fresh agar-solidified plates, and incubated for another 5 days at 30 °C. The step was repeated two times. Separated single colonies in plates were further amplified and sequenced at Sangon Biotech Co., Ltd (Shanghai, China). Subsequently, 16S rDNA sequences were aligned and analyzed based on sequences registered in GenBank using BLAST (v2.13.0; https://blast.ncbi.nlm.nih.gov/Blast.cgi). All purified isolates were cultured in 20-ml screening medium at 30 °C with shaking (180 rpm) for 24 h before freezing and preservation at − 80 °C in 30% glycerol (v/v) for future use.

### Genome-wide sequencing of isolates

After activation and centrifugation, four isolates were used to perform DNA extraction and genome-wide sequences based on an Illumina PE150 platform (Illumina). Gene detection of the assembled genomes was carried out using RAST web server (https://rast.nmpdr.org/). To illustrate the overall metabolic capability of those four isolates, the function of genes encoded by the isolates was predicted using the KEGG KofamKOALA tool [80] and classified using COG categories. To explore functional differences and similarity among isolates, their pan-genome was constructed using Orthofinder2 software with the default parameters [81]. The core ortholog groups were further manually checked based on the functional assignment. Additionally, two specific genes identified in pan-genome analysis for each isolate were selected for use in the design of the specific primers for quantification of inoculant colonization. The primers were detected using Primer premier v5 [82].

### Degradation and colonization ability of inoculants

The allelochemical (glycyrrhizin) degradation efficiency and metabolites in pure culture and rhizosphere soil were analyzed using high-resolution ion mobility liquid mass spectrometry (AB SCIEX, Boston, USA), as described in other studies [2, 23, 37]. First, inorganic salt solution containing glycyrrhizin (10 g l^−1^) as the sole carbon source was used for evaluating the ability of the isolates to degrade glycyrrhizin and corresponding metabolites in pure culture. Proportionate amounts of NaOH were also added to the culture solutions to adjust the pH (7.5–8.0), with six replicates for each isolate and synthetic inoculant treatment. An isolated colony of each rhizobacterium and synthetic inoculant was used to inoculate 20 mL of solution cultured at 30 °C with shaking (180 rpm). After incubation for 5 days, the cultured solutions were extracted with ethyl acetate, and the extraction liquid was dried using a vacuum rotary evaporator at room temperature (25 °C). The residue was replenished with 100% methanol (MeOH) and then passed through a 0.45-μm nylon membrane filter for use in liquid chromatography tandem mass spectrometry (LC–MS/MS) analysis. Secondly, glycyrrhizin in rhizosphere soil was extracted and analyzed. Briefly, a sieved sample (11 g) was extracted by MeOH (60 mL) with sonication 2 times (30 min each time), and then centrifugated at 6000 rpm for 5 min. The supernatant was filtered and then concentrated in vacuum to near dryness on a rotary evaporator. Subsequently, the residue was redissolved in MeOH and passed through a 0.45-μm nylon membrane filter prior to LC–MS/MS analysis. The details of LC–MS/MS analysis have been described previously in another study [83].

Total RNA was extracted from 0.5-g rhizosphere soils using an RNA PowerSoil® Total RNA Isolation kit (MOBio, Qiagen, USA). Purified RNA and complementary DNA (cDNA) were obtained according to the above manufacturer’s protocol. To explore the colonization of rhizobacterial inoculants, the real-time absolute abundance of inoculants in each sample was quantified by quantitative nested real-time PCR (qNRT-PCR) [84, 85] using a Quantstudio 6 Flex real-time PCR system (Thermo Fisher, Carlsbad, CA, U.S.A.) and SYBR Premix Ex Taq II (TaKaRa). The method enhanced the sensitivity of the assay, because inoculant RNA detection in the rhizosphere soil could be challenging considering their low concentrations. In general, thermal cycling conditions were as follows: an initial denaturation phase at 95 °C for 10 min; first-round PCR (outer PCR) followed by 10 cycles at 95 °C for 30 s, 55 °C for 30 s, and 72 °C for 30 s; and second-round PCR (inner PCR) followed by 40 cycles at 95 °C for 30 s, 58 °C for 30 s, and 72 °C for 40 s. Data collection was performed at 72 °C. In addition, a melting curve was generated to monitor amplification specificity. The nested primers developed in the present study were specific and sensitive to detect inoculants in rhizosphere soil and are listed in Table S2. qNRT-PCR analysis was carried out with independent biological replicates and three technical replicates. Additionally, plasmid DNAs were diluted to yield a series of concentrations with tenfold differences for use in the generation of a standard curve. For total bacterial 16S rRNAs, standard plasmids were prepared in the 5.98 × 10^2^–5.98 × 10^9^ copies range. The efficiencies of the qNRT-PCR assays ranged from 90 to 110% and the *R*^2^ value for each standard curve line exceeded 0.98. The Ct value (threshold cycle) was determined to quantify the copy number of specific inoculants.

### Statistical analyses

All statistical analyses were conducted in the R environment (v3.6.3; http://www.r-project.org/). Most of the results were visualized using the “ggplot2” package [86], unless otherwise indicated. Additionally, all of the *P* values were adjusted using the false discovery rate method [87]. Plant performance (chlorophyll content, fresh shoot, and root weights), soil characteristics (properties and enzyme activity), expression profiles of major secondary metabolite synthesis genes, microbial alpha diversity indices, and inoculant colonization of rhizosphere soil were compared using one-way analysis of variance (ANOVA), followed by comparisons of means using Tukey’s HSD parametric tests (“multcomp” package) [88]. Principal coordinate analysis (PCoA) was performed using the “Ape” package [89] and significant differences in bacterial community composition were tested using Permutational Multivariate Analysis of Variance (PERMANOVA) using the “Adonis” function in the “Vegan” package [90] based on Bray–Curtis distances. Analysis of the differently enriched rhizobacterial taxa was conducted using significant difference analyses using the “edgeR” package [91]. Persistent taxa were selected from enriched taxa overlapping across different stages and treatments in Venn diagrams generated using the “Venndiagram” package [92]. A Random Forest (RF) classification model was used to identify bioindicators between different treatments using the “rfPermute” package [93]. Cross-validation was performed to select appropriate features (taxa) [33]. Subsequently, phylogenetic trees of the enriched taxa were constructed using the “ggtreeExtra” package [94]. Heatmaps were illustrated based on *Z*-score-normalized relative abundances of taxa using the “pheatmap” package [95] and were attached to the phylogenetic trees. Co-occurrence networks were constructed based on the relationships among the persistent taxa using Spearman correlations (|*r*|> 0.8, *P* < 0.01) (“igraph” package) [96]. The network was visualized using the “Gephi” interactive platform [97]. The top five taxa with high betweenness centrality values were considered keystone species [98]. In addition, the nodes were defined as network hubs, module hubs, connectors, and peripherals based on the threshold of Zi-score (2.5) and Pi-score (0.62) values [99].

## Supplementary Information

**Additional file 1: Table S1.** PCR primers used in this study. *HMGR*, 3-hydroxy-3-methylglutary coenzyme A reductase gene; *β-AS*, bamyrin synthetase gene;* CYP88D6* and *CYP72A154*, cytochrome P450 monooxygenases gene;* LUS*, lupeol synthase gene;* CHS*, chalcone synthase gene; *β-actin* and *18s rRNA* reference gene. **Table S2.** Quantitative nested real-time PCR (qNRT-PCR) primers of inoculants. **Table S3.** Allelochemical content in rhizosphere soil after distinct inoculants. **Table S4.** Screened pangenomes related to housekeeping functions of four isolates. **Fig. S1.** The network (a) and Zi-Pi plot (b) composed of persistent taxa based on Spearman correlation method. **Fig. S2.** Plate confrontation experiment between colonies of E (*Ensifer sesbaniae*) and N (*Novosphingobium resinovorum*) inoculants. **Fig. S3.** Bar plots of gene copy numbers of colonization of rhizobacterial inoculants under different inoculants and exogenous glycyrrhizin addition. I, initial sampling stage; A, allelochemical treatment; W, water treatment; C, control: no inoculants, N, *Novosphingobium resinovorum* inoculants; E, *Ensifer sesbaniae* inoculants; S, synthetic inoculants. Different letters indicate significant differences (*P* < 0.05; One-way ANOVA, Tukey’s HSD test).

## Data Availability

The datasets used and/or analyzed during the current study are available from the corresponding author on reasonable request.
