# Supplementary material for: Metagenomics insights into responses of rhizobacteria and their alleviation role in licorice allelopathy
Source: Microbiome. 2023 May 22;11:109. doi: 10.1186/s40168-023-01511-3 (PMC10201799; doi:10.1186/s40168-023-01511-3)
Supplement: Supplementary file 2 — Additional file 1: Table S1. PCR primers used in this study. HMGR, 3-hydroxy-3-methylglutary coenzyme A reductase gene; β-AS, bamyrin synthetase gene; CYP88D6 and CYP72A154, cytochrome P450 monooxygenases gene; LUS, lupeol synthase gene; CHS, chalcone synthase gene; β-actin and 18s rRNA reference gene. Table S2. Quantitative nested real-time PCR (qNRT-PCR) primers of inoculants. Table S3. Allelochemical content in rhizosphere soil after distinct inoculants. Table S4. Screened pangenomes related to housekeeping functions of four isolates. Fig. S1. The network (a) and Zi-Pi plot (b) composed of persistent taxa based on Spearman correlation method. Fig. S2. Plate confrontation experiment between colonies of E (Ensifer sesbaniae) and N (Novosphingobium resinovorum) inoculants. Fig. S3. Bar plots of gene copy numbers of colonization of rhizobacterial inoculants under different inoculants and exogenous glycyrrhizin addition. I, initial sampling stage; A, allelochemical treatment; W, water treatment; C, control: no inoculants, N, Novosphingobium resinovorum inoculants; E, Ensifer sesbaniae inoculants; S, synthetic inoculants. Different letters indicate significant differences (P < 0.05; One-way ANOVA, Tukey’s HSD test). [file 40168_2023_1511_MOESM1_ESM.zip › Supplementary Material.docx]

**Metagenomics insights into responses of rhizobacteria and their alleviation role in licorice allelopathy**

**Running title:** Rhizobacteria alleviate licorice allelopathy

**Yang Liu ^a1^,** **Hao Wang ^a1^,** **Xun Qian^bc^, Jie Gu^bc^, Weimin Chen^a^, Xihui Shen^a^, Shiheng Tao^a^, Shuo Jiao ^a^*,** **Gehong Wei ^a^***

**^a^***State Key Laboratory of Crop Stress Biology for Arid Areas, Shaanxi Key Laboratory of Agricultural and Environmental Microbiology,* *College of Life Science, Northwest A&F University, Yangling, Shaanxi 712100, People's Republic of China*

**^b^***Interdisciplinary Research Center for Soil Microbial Ecology and Land Sustainable Productivity in Dry Areas, Northwest A&F University, Yangling 712100, Shaanxi, China.*

**^c^***College of Natural Resources and Environment, Northwest A&F University, Yangling 712100, Shaanxi, China.*

*Corresponding authors. State Key Laboratory of Crop Stress Biology for Arid Areas, Shaanxi Key Laboratory of Agricultural and Environmental Microbiology, College of Life Science, Northwest A&F University, 3 Taicheng Road, Yangling, Shaanxi 712100, People's Republic of China.

Tel.: +86 29 87091175; Fax: +86 29 87091175; *E-mail addresses:* weigehong@nwsuaf.edu.cn (G. Wei) and shuojiao@nwsuaf.edu.cn (S. Jiao).

^1^ These authors contributed equally to this work.

The supporting information includes four tables and three figures.

**Summary**

**Table S1** PCR primers used in this study. *HMGR*, 3-hydroxy-3-methylglutary coenzyme A reductase gene; *β-AS*, bamyrin synthetase gene; *CYP88D6* and *CYP72A154*, cytochrome P450 monooxygenases gene; *LUS*, lupeol synthase gene; *CHS*, chalcone synthase gene; *β-actin* and *18s rRNA* reference gene.

**Table S2** Quantitative nested real-time PCR (qNRT-PCR) primers of inoculants.

**Table S3** Allelochemical content in rhizosphere soil after distinct inoculants.

**Table S4** Screened pangenomes related to housekeeping functions of four isolates

**Fig. S1** The network **(a)** and Zi-Pi plot **(b)** composed of persistent taxa based on Spearman correlation method.

**Fig. S2** Plate confrontation experiment between colonies of E (*Ensifer sesbaniae*) and N (*Novosphingobium resinovorum*) inoculants.

**Fig. S3** Bar plots of gene copy numbers of colonization of rhizobacterial inoculants under different inoculants and exogenous glycyrrhizin addition. I, initial sampling stage; A, allelochemical treatment; W, water treatment; C, control: no inoculants, N, *Novosphingobium resinovorum* inoculants; E, *Ensifer sesbaniae* inoculants; S, synthetic inoculants. Different letters indicate significant differences (*P* < 0.05; One-way ANOVA, Tukey’s HSD test).

**Table S1** PCR primers used in this study. *HMGR*, 3-hydroxy-3-methylglutary coenzyme A reductase gene; *β-AS*, bamyrin synthetase gene; *CYP88D6* and *CYP72A154*, cytochrome P450 monooxygenases gene; *LUS*, lupeol synthase gene; *CHS*, chalcone synthase gene; *β-actin* and *18s rRNA* reference gene.

| Genes | Accession No. | Forward (5’→3’) | Reverse (5’→3’) | Fragment length (bp) |
| --- | --- | --- | --- | --- |
| *HMGR* | JF461267.1 | GGTAACAGCTCCCTCTCCAC | CCCGTTATCCTCTGCAC | 192 |
| *β-AS* | GU072921.1 | TGCGAATTCTTGGAGAAG | AGGATCCAAAACTCAGG | 181 |
| *CYP88D6* | AB433179.1 | CAACCCGTTGTGGATGAAAG | ATGGCCAGCGAACAAAA | 168 |
| *CYP72A154* | AB558153.1 | GAACACGCTGTGGCTGAG | GATGGGCAAGGGAGAAGA | 194 |
| *LUS* | AB663343.1 | CATTACGAGGACGAGAACAG | ATCAGGGATTCGGGCTAA | 196 |
| *CHS* | HQ840673.1 | AAAGCTCTTGGGCCTTC | TCAAGATGAGTGTCAGTGG | 186 |
| *β-actin* | EU190972.1 | CCAGTGCTTCTAACTGAG | CAATACCAGTTGTACGA | 150 |
| *18s rRNA* | NG_065099.1 | CGGCGACAGAAGGGACGAGACGA | GGTGGTGACGGGTGACGGAGAAT | - |

**Table S2** Quantitative nested real-time PCR (qNRT-PCR) primers of inoculants.

| Inoculants | Outer primer-F (5’→3’) | Outer primer-R (5’→3’) | Inner-sense (5’→3’) | Inner-antisense (5’→3’) |
| --- | --- | --- | --- | --- |
| *Ensifer sesbaniae* (E) | GGCAAGACACGCAGTTATCG | GAAACGGTCGGGTTCAGG | ATGTTCCGATTTATCCA | TCATGGAGTAATCGCCTGTG |
| *Novosphingobium resinovorum* (N) | ACGCTGACTTTCGGAGGC | GTACGACCGCTGAGGCAAT | ATGACGCAGCGCCTTCAATCTTCCG | TCAAGCAGCGCCGGTGGC |

**Table S3** Allelochemical content in rhizosphere soil after distinct inoculants.

| Treatment | Inoculation | Allelochemical content (%) | Relative degradation rate (%) |
| --- | --- | --- | --- |
| A | C | 58.44 | - |
|  | N | 34.94 | 40.21 |
|  | E | 54.54 | 6.67 |
|  | S | 39.16 | 32.99 |
| W | C | 52.28 | - |
|  | N | 32.36 | 38.10 |
|  | E | 20.19 | 61.38 |
|  | S | 36.73 | 29.74 |

Note: A, allelochemical treatment; W, water treatment; C, control: no inoculants, N, *Novosphingobium resinovorum* inoculants; E, *Ensifer sesbaniae* inoculants; S, synthetic inoculants.

**Table S4** Screened pangenomes related to housekeeping functions of four isolates

**Table S4** is available online as a separate .CSV file under the Supplemental information for this article


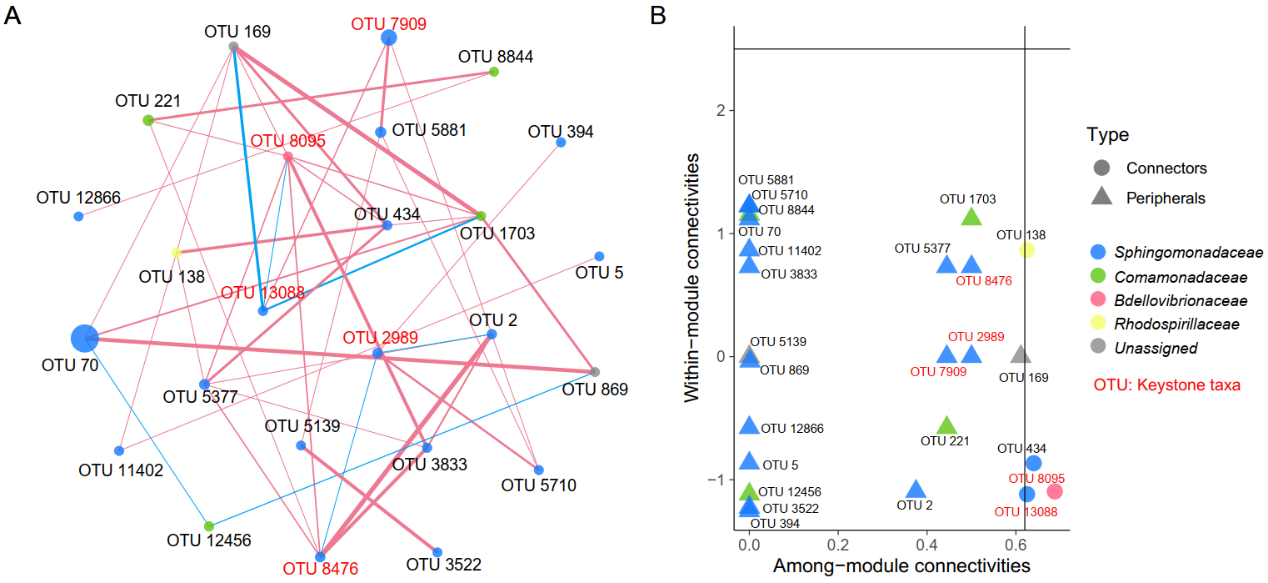
**Fig. S1** The network **(a)** and Zi-Pi plot **(b)** composed of persistent taxa based on Spearman correlation method.


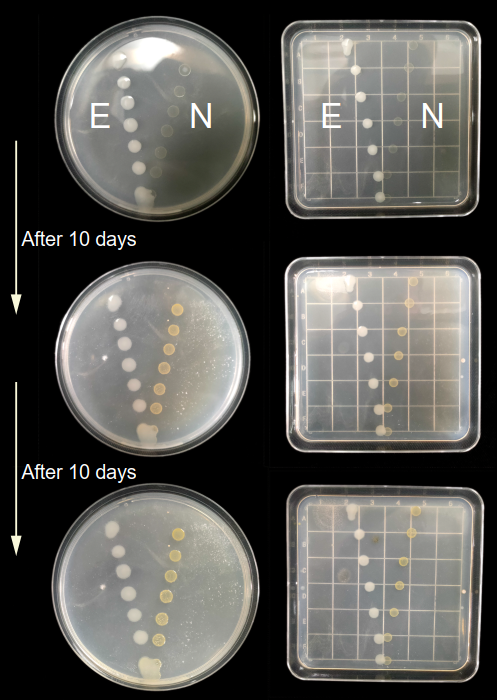
**Fig. S2** Plate confrontation experiment between colonies of E (*Ensifer sesbaniae*) and N (*Novosphingobium resinovorum*) inoculants.


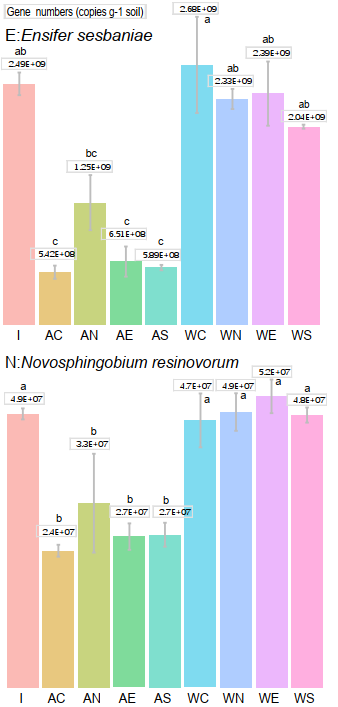
**Fig. S3** Bar plots of gene copy numbers of colonization of rhizobacterial inoculants under different inoculants and exogenous glycyrrhizin addition. I, initial sampling stage; A, allelochemical treatment; W, water treatment; C, control: no inoculants, N, *Novosphingobium resinovorum* inoculants; E, *Ensifer sesbaniae* inoculants; S, synthetic inoculants. Different letters indicate significant differences (*P* < 0.05; One-way ANOVA, Tukey’s HSD test).
